# Balancing the dual nature of glycotoxins: interplay of diet, digestion, and gut microbiome

**DOI:** 10.3389/fnut.2025.1693167

**Published:** 2025-11-28

**Authors:** Halyna Semchyshyn

**Affiliations:** Department of Biochemistry and Biotechnology, Vasyl Stefanyk Carpathian National University, Ivano-Frankivsk, Ukraine

**Keywords:** glycation, advanced glycation end products, reactive carbonyl species, diet, digestion, gut microbiome

## Abstract

Glycation chemistry, both *in vitro* and *in vivo*, is well-studied and known to result in a variety of products—from early glycation products, reactive carbonyl and oxygen species (RCS and ROS, respectively) to advanced glycation end products (AGEs). Exogenous glycation products from the regular diet contribute substantially to the total AGE burden, often exceeding endogenous formation. AGEs and other products of glycation, whether formed endogenously or derived exogenously, may have similar biological effects and are mainly known for their harmful impact, therefore, the term “glycotoxins” is used to emphasize the toxicity of certain of them. Nevertheless, the human body as well as gut microbiome have adapted to the presence of glycation products and can even use them beneficially at low concentrations. Maintaining an appropriate balance of glycotoxins depends largely on digestion in the gastrointestinal tract, mediated by both host and microbiome enzymes. The fate of dietary glycation products in the gut strongly depends on their interaction with the intestinal microbiota. A key open question is how human and microbial enzymes work together to degrade AGEs and maintain their concentrations within a potentially “beneficial” range. This review is focused on the metabolism and digestion of glycation products by both human and microbial enzymes, highlighting their dual nature and overall impact on human health.

## 1 Introduction

Western diet is the dominant dietary pattern worldwide and is associated with chronic health disorders, sometimes referred to as “Western diseases.” One of the main features of the Western dietary pattern is excessive intake of processed foods and added sugars, which creates favorable conditions for the Maillard reaction (1–4). Glycation is another term often used in relation to the Maillard reaction, however they are not the same. Actually, glycation is a complex net of nonenzymatic interactions, initiated by the Maillard reaction—an interaction between reducing sugar and some nucleophile (*e.g.*, amino acid or protein). Like free-radical chain reactions, glycation process is characterized by multiple steps with an unpredictable course and a wide variety of intermediates and end products. This complexity is one of the reasons for the widespread use of the term “Maillard chemistry” to describe the broad character of glycation processes (5–7). In living organisms, glycation is a part of normal metabolism and can lead to irreversible modifications of biomolecules and damage to cellular constituents, resulting in the formation of a variety of poorly degradable, heterogeneous compounds collectively known as advanced glycation end products (AGEs). Glycation also commonly occurs during food processing and storage (8), producing AGEs *in vitro* that we consume with food; in this context, they are referred to as dietary AGEs (dAGEs).

Glycation products, whether endogenous or exogenous, exert similar effects in the organism, which are largely detrimental; therefore, they are often referred to as glycotoxins and are particularly implicated in the development of noncommunicable chronic diseases, especially those associated with inflammation (2, 3, 9–15). To mitigate the potentially harmful consequences of AGE accumulation, organisms have evolved a range of protective mechanisms. They include enzymatic detoxification systems, receptor-mediated clearance, and proteolytic degradation. Among these, the digestion of dietary glycotoxins, and dAGEs in particular, in the gastrointestinal tract—mediated by both host and microbiome enzymes—appears to be a potentially important and poorly investigated aspect of the defense against AGE-related damage. In addition, the human body as well as gut microbiome have adapted to the presence of glycation products and can even use them—but only at low “beneficial” concentrations. Determining the physiological range of glycotoxins in the organism remains an unresolved issue.

This review explores some aspects of the metabolism and digestion of glycotoxins by both human and microbial enzymes, highlighting how their interplay can balance the dual nature of these compounds and their impact on human health.

### 1.1 A brief look at Maillard chemistry

The nonenzymatic reaction between monosaccharides and amino acids was first described by Louis Camille Maillard in 1912 (16). About 40 years later, the Maillard reaction was recognized as one of the main causes of nonenzymatic browning of food, highlighting its importance in food science and technology (5, 17). In the late 1960s, products of nonenzymatic glycosylation similar to food browning products were detected in the human body (18, 19). It took several decades to appreciate the physiological significance of the reaction described by Maillard, which has since attracted renewed attention in biochemistry and medicine. The nonenzymatic glycosylation process was named “glycation” to distinguish it from enzymatic glycosylation—an important post-translation modification of proteins (20). In the 1980s, “glycation hypothesis of aging,” which postulates that glycation plays a causative role in aging and age-related pathologies, was formulated (21, 22). Today, this theory continues to support the growing interest in the field of *in vivo* glycation and its relevance to normal and pathological age-related processes.

**Figure 1:**
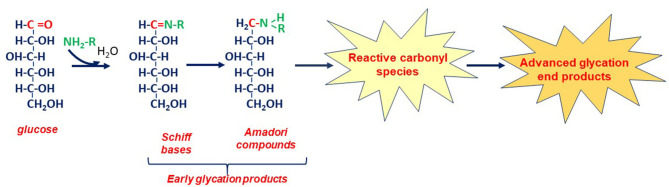
Involvement of glucose in the nonenzymatic process—glycation. The initial step of glycation (the Maillard reaction) is a covalent interaction between the electrophilic carbonyl group of open-chain glucose and the nucleophilic functional group of a biomolecule (*e.g*., the amino group of an amino acid). This interaction produces a range of early glycation products—Schiff bases and Amadori products. Unstable Schiff bases undergo isomerization to form more stable Amadori adducts. The glucose moiety of the Amadori products can further undergo enolization, followed by dehydration, oxidation, and/or fragmentation reactions, consequently producing a variety of reactive carbonyl species (RCS). In the advanced stages of glycation, these early products and reactive species further undergo a complex of irreversible nonenzymatic reactions, ultimately producing a broad spectrum of advanced glycation end products (AGEs).

The initial step of glycation is the Maillard reaction (Figure 1)—a covalent interaction between the carbonyl group of glucose or another reducing carbohydrate and an amino group (or other nucleophilic functional group) of a biomolecule, resulting in the Schiff base formation. Such bases are rather unstable and convert into more stable early glycation products known as Amadori compounds. They, in turn, undergo oxidation, rearrangements, and fragmentation reactions, producing a range of reactive carbonyls. These intermediates are highly reactive: if glycation can be compared to free radical chain reactions, RCS can be paralleled with ROS (23). Reactive carbonyls, typically low-molecular-mass compounds containing three to nine carbons and one or more carbonyl groups, readily react with nucleophilic groups of proteins, lipids, and nucleic acids, leading to the formation of AGEs. The latter are able to generate both RCS and ROS, thereby initiating additional rounds of glycoxidation and creating vicious nonenzymatic cycles that further increase glycotoxin concentrations and contribute to development of carbonyl/oxidative stress (23–30). This progression from early glycation products to RCS and ultimately to AGEs represents an important part of the pathway of nonenzymatic glycation both *in vivo* and during food processing.

### 1.2 Metabolism of carbohydrates and their relation to the generation of glycation products

Under physiological conditions, carbohydrates such as glucose or fructose are considered among the major contributors to glycation process. Importantly, free carbonyl group in open-chain monosaccharides can directly participate in the nonenzymatic process (Figure 1).

**Figure 2:**
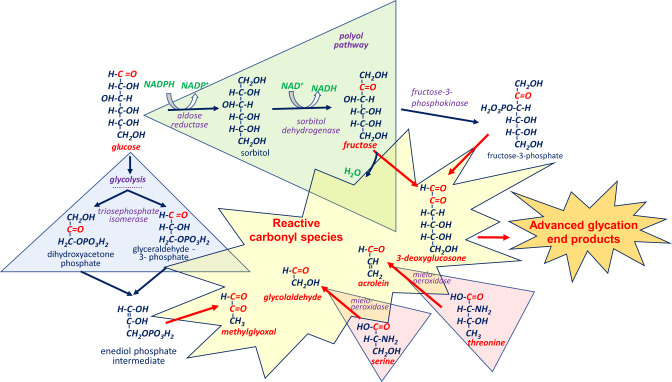
Involvement of glucose, fructose, and amino acids in the enzymatic processes, producing reactive carbonyl species (RCS) and advanced glycation end products (AGEs). *Glycolysis*: Glucose enters glycolytic pathway, where such RCS as methylglyoxal can be generated at the stage of triosephosphate isomerase reaction. *Polyol pathway*: Glucose, entering polyol (sorbitol) pathway, is first reduced to sorbitol by aldose reductase and then oxidized to fructose by sorbitol dehydrogenase. Fructose can subsequently be converted to such RCS as 3-deoxyglucosone either through fructose dehydration or *via* reaction catalyzed by fructose-3-phosphokinase. *Amino acid oxidation*: Serine and threonine are oxidized by myeloperoxidase to form such RCS as glycolaldehyde and acrolein, respectively.

Enzymatic metabolism can also contribute to Maillard chemistry (Figure 2), although not necessarily directly. Among such processes, glycolysis and polyol pathway are probably the best studied examples, as they are well-known sources of carbohydrate-derived RCS. Depending on the conditions, ~0.1%−0.4% of glycolytic intermediates can be converted to the side-product methylglyoxal, which belongs to α-dicarbonyl compounds—one of the most reactive RCS (31, 32). The proposed explanation is that enediol phosphate intermediate of the triosephosphate isomerase reaction may escape from the enzyme's active site and rapidly converted to methylglyoxal (32–35). Consistent with this, inactivation of glycolytic enzymes—triosephosphate isomerase or glyceraldehyde-3-phosphate dehydrogenase—elevates the level of upstream intermediates, thereby enhancing methylglyoxal generation (11).

The polyol pathway is another important contributor to RCS and AGE pool, that becomes activated under hyperglycemic conditions. In this pathway, glucose is first reduced to sorbitol by aldose reductase and then oxidized to fructose by sorbitol dehydrogenase. Fructose can subsequently be converted to 3-deoxyglucosone by either its dehydration or reaction catalyzed by fructose-3-phosphokinase (25). In addition, fructose itself initiates glycation more actively than glucose (6, 36–40).

Under inflammatory conditions, RCS can also be enzymatically generated by activated human phagocytes. For example, it has been shown that stimulated neutrophils employ myeloperoxidase to produce aldehydes from hydroxy-amino acids (41).

Reactive carbonyls appeared in either nonenzymatic or enzymatic reactions can react with nucleophilic groups of biomolecules, leading to further formation of AGEs. It should be noted that *in vivo* formation of glycation products is a relatively slow process with delayed biological effects. In contrast, exposure to exogenous AGEs might have faster effects.

### 1.3 Absorption of dietary glycotoxins and their dual biological impacts

**Figure 3:**
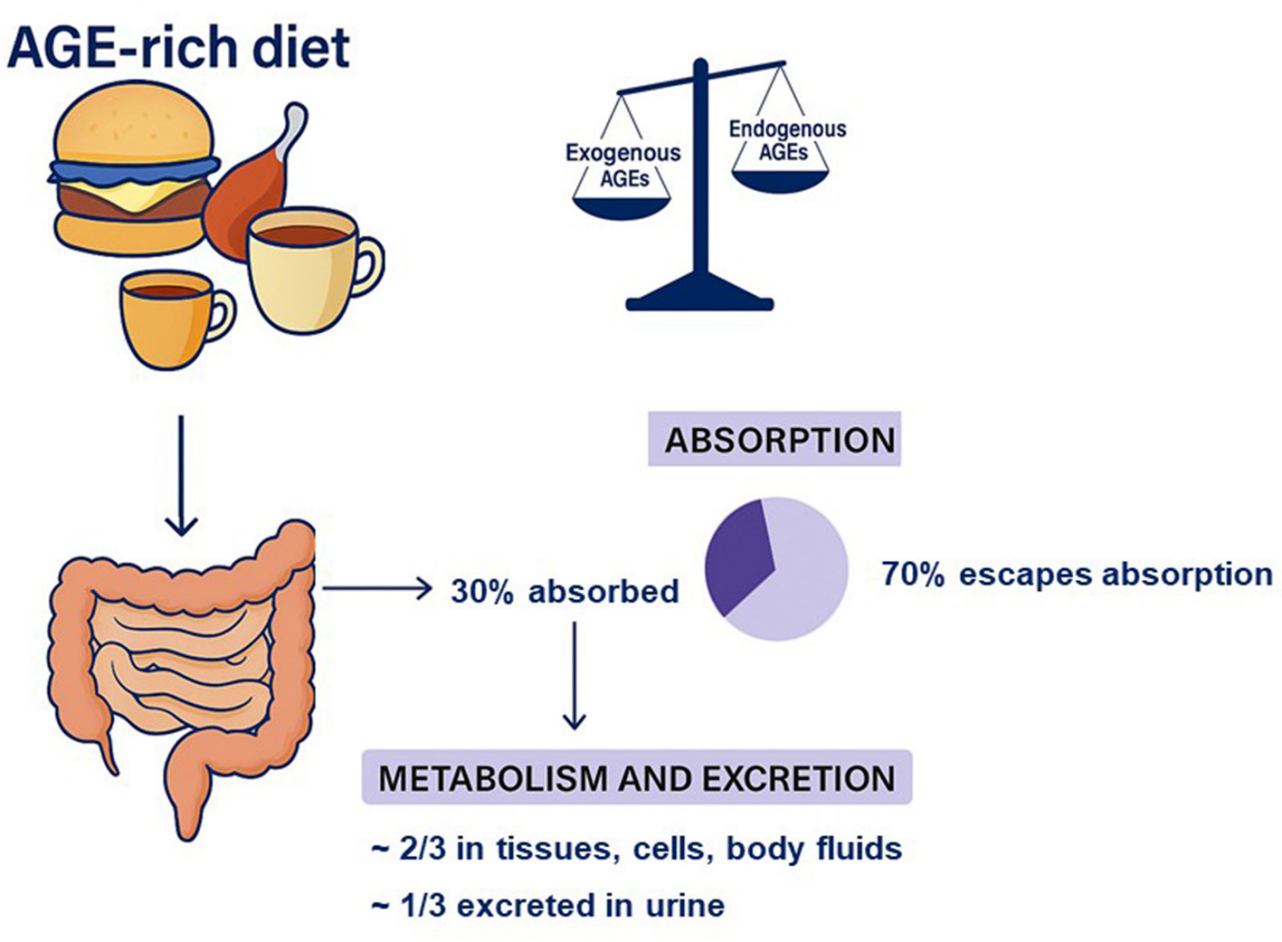
Metabolic fate of dietary advanced glycation end products (dAGEs) from foods typical of a Western diet. Exogenous glycation products derived from the daily diet can exceed their endogenous formation and contribute substantially to the total AGE burden in the human organism, making the Western diet an important external source of these compounds. dAGEs enter the digestive system, where up to 30% are absorbed in the gastrointestinal tract, while approximately 70% survive the initial stages of digestion and therefore reach the colon, where they interact with the gut microbiota. Of the absorbed fraction, about two-thirds remain distributed in tissues, cells, and body fluids, while about one-third is excreted.

Besides endogenous RCS and AGEs, exogenous glycation products, derived from the regular diet, contribute substantially to the total AGE burden in the human organism (Figure 3). Most foods naturally contain dietary glycation products, and dAGEs in particular, often in amounts that can exceed their endogenous generation, making the daily diet a major source of glycotoxins (13, 24, 42, 43). Several comprehensive studies have quantified dAGE content in commonly consumed foods, providing a valuable basis for developing databases of dAGEs (12, 13, 44–49). However, the lack of standardized analytical methods for measuring glycation products remains a significant limitation for the direct comparison of data across laboratories.

To date, approximately forty distinct AGEs have been identified in various materials—from foods to human fluids and tissues (3, 8, 50–52). These compounds represent a large and heterogeneous group differing in their sources, molecular masses, physicochemical properties, and cross-linking abilities. For example, many of them are fluorescent crosslinks (*e.g.*, pentosidine), non-fluorescent crosslinks (*e.g.*, glyoxal- and methylglyoxal-lysine dimers, known as GOLD and MOLD, respectively), or non-fluorescent and non-crosslinking adducts (*e.g.*, carboxymethyllysine (CML), carboxymethylcysteine, and argpyrimidine). In addition to their diverse properties, AGEs are associated with a wide range of biological effects. These complexities have prompted the development of various analytical methods for their identification and quantification. Among the most frequently employed approaches are fluorimetric detection, immunochemical assays, determination of individual targetes using selective analytical techniques, and explorative studies aimed at the structural characterization of identified AGE species (47, 48, 53). However, considerable discrepancies exist among laboratories in AGE quantification results. These differences arise at least from the lack of standardized analytical protocols and reference materials, as well as variations in calibration procedures, and differences in antibody specificity or detection principles.

#### 1.3.1 Glycotoxins in foods and their absorption

Despite the abovementioned, available data demonstrate that the levels of dAGEs vary widely among food groups and cooking methods. It should be noted that dAGEs are naturally present even in raw, unprocessed foods and therefore cannot be completely avoided. However, different foods contribute differently to total dAGE intake. For example, processed nuts, baked goods, certain meats, and cereals often contain higher levels of dAGEs (>150 mg/kg) than dairy products, vegetables, and fruits (< 40 mg/kg) (49). It is also well-demonstrated that concentrations of glycation products can increase dramatically with food processing, particularly with high-temperature cooking. Such techniques as frying, broiling, grilling, and roasting significantly promote dAGE formation, while steaming, stewing, poaching, and boiling generally yield comparatively lower amounts. For instance, poached or steamed chicken meat has less than one-fourth the dAGE content of roasted or broiled chicken meat; raw carrots have 2.5- and 22-fold lower dAGE levels than boiled and grilled carrots, respectively (46). Some popular beverages are also notable sources of glycotoxins, and preparation methods matter: drip coffee contains about 1.6 AGE kU/100 ml, whereas instant coffee has about 5 kU/100 ml; fruit tea contains about 0.4 AGE kU/100 ml, while tea prepared from tea bags can reach 2 AGE kU/100 ml (46).

Consumption of precursors for AGEs with a regular diet also influences the amounts of glycotoxins in the human body. For example, the daily intake of Amadori products (calculated as fructoselysine) has been estimated at 500–1,200 mg per day, whereas typical consumption of dAGEs (calculated as pyrraline and carboxymethyllysine) has been estimated ~25–75 mg daily (54, 55). High-fructose corn syrup, widely used as a sweetener in processed foods and beverages, is a major source of RCS such as 3-deoxyglucosone, glyoxal, and methylglyoxal, providing precursors for AGE formation (42, 56, 57). In fact, dAGEs and their precursors are unavoidable components of the human diet, and the Western dietary pattern—characterized by high intake of processed and sweetened products—is strongly associated with high exposure to dietary glycotoxins.

An important question is to what extent dAGEs are absorbed (Figure 3). Human studies suggest that about 70% of ingested dAGEs escape absorption, while up to 30% survive digestion and are absorbed through the gastrointestinal tract, then appearing in the circulation as glycotoxins in amounts proportional to dietary intake (58–64). Of the absorbed dAGEs, about one-third is excreted in urine, whereas the remaining fraction is likely distributed via the bloodstream and retained within tissues, cells, and body fluids. Consuming an AGE-rich diet—with dAGE levels approximately three times higher than those in a standard diet—has been shown to increase circulating AGE concentrations by about 50% (62). Conversely, when a diet low in dAGEs is consumed, urinary excretion of pentosidine can decrease by about 40% and excretion of pyrraline and fructoselysine can drop by about 90% (61). Interestingly, plasma AGE concentrations have been found to be significantly higher (by up to 25%) in vegetarians compared with omnivores. Among dietary groups, vegans, lacto-ovo vegetarians, and semi-vegetarians show plasma AGE levels that are by 15, 32, and 22% higher, respectively, than those in omnivores (65, 66).

It should be noted that strong positive correlations, such as those mentioned above, are not consistently observed across all studies. This likely reflects the complexity of the relationship between dAGE intake and AGE levels in the body, which depends on multiple factors, including the type of diet, the specific kinds of dAGEs consumed, and an individual's physiological state. Nevertheless, although a large portion of ingested dAGEs escapes absorption and about 10% of those absorbed are excreted, the fraction that remains can exert significant biological effects, which, according to most studies, are detrimental.

#### 1.3.2 Dual biological effects of glycotoxins

Glycation products exert diverse effects through their capacity to generate reactive species, that can contribute to damage and dysfunction of biomolecules (13, 25). Furthermore, ingested dAGEs may have toxicological effects by changing or even disrupting the essential gut-friendly microbiome (10, 44, 67, 68). Both dietary and endogenous AGEs can activate cell signaling pathways by binding to specific receptors such as the receptor for advanced glycation end products (RAGE). This activation promotes various downstream effects, including inflammation, proliferation, apoptosis, autophagy and other processes associated with many human pathologies such as diabetes, neurodegenerative and cardiovascular diseases (13, 23, 24, 69–72). Importantly, RAGE is not specific only to AGEs; it can bind multiple ligands, initiating a complex RAGE signaling network that is not yet fully understood. Therefore, “negative” effects of RAGE expression cannot always be attributed solely to AGEs.

Although glycotoxins are primarily known for their detrimental effects, certain mechanisms are involved in both their deleterious and beneficial effects. It can be assumed that long-term exposure to these compounds has driven adaptative response leading to mechanisms that may confer specific benefits. The dual nature of glycotoxins appears to be dose- and time-dependent. Such benefits, at least in part, may arise from AGE-induced RAGE expression at normal physiological levels, which provides several essential functions such as modulating the immune response and cell differentiation, supporting tissue regeneration and resistance against cell death (72–74). Some authors suggest that, in homeostatic systems free of chronic inflammation, hyperglycemia, or sustained oxidative stress, basal RAGE expression serves as an important first line of defense against infection, inflammation, or injury (72). However, under conditions of imbalance, which can be caused by elevated levels of RAGE ligands, including either endogenous or dietary AGEs, this scenario may shift toward the development of chronic carbonyl/oxidative stress and related diseases. This likely why the majority of available data still mainly focus on the harmful effects of RAGE activation.

Another example of potential benefits from some glycotoxins is a mild/temporary stress (in contrast to the chronic stress) induced by low doses and/or short exposure period. Such mild carbonyl/oxidative stress underlies phenomena including preadaptation, cross-adaptation, and hormesis, that can strengthen the cell's response and survival under severe and even lethal stressful conditions (75–81). In addition, different glycation intermediates demonstrate antibacterial, antiprotozoal, antifungal, antiviral, and anticancer activities. Notably, some of them are naturally occurring compounds. For example, methylglyoxal and glyoxal have been found in New Zealand Manuka honey that is known for its very high antibacterial activity. Methylglyoxal was detected at concentrations, which is up to 100-fold higher compared to conventional honey (82).

Some glycotoxins have been demonstrated to alter cell signaling and gene expression (23, 24, 33, 83–88). The understanding of glycation products as mediators of intracellular signaling has advanced considerably over the past decades. However, most available data concern their detrimental effects and the mechanisms involved in the development of pathologies, while many aspects remain unclear and require further investigation. Earlier discussions focused on whether certain glycation products meet the general criteria for signaling molecules, which are characterized by: (i) regulated intracellular levels, (ii) sufficient stability and ability to reach their targets, (iii) specific receptor interactions that initiate downstream responses, and (iv) reversibility of their effects. Among the diverse group of glycotoxins, some generally meet some of these criteria. For instance, RCS and other low-molecular-mass glycation products fulfill several requirements. First, their formation and elimination are at least partially enzyme-controlled and they can diffuse across membranes. For example, methylglyoxal, glyoxal, 3-deoxyglucosone and other glycation agents are produced by methlglyoxal synthase, hydroxyacid oxidase, enzymes of polyol pathway (25, 31, 32). Next, low molecular mass, noncharged structure, and relatively high stability of RCS allow them to cross hydrophobic biological membranes, diffuse over considerable long distances in the hydrophilic intracellular environment, and even to escape from the cell to interact with targets distant from their sites of formation (89–93). Finally, glycation products interact with specific receptors, including isoforms of mentioned above multifaceted RAGE and its counterparts such as AGE-R1, AGE-R2, and AGE-R3, which are involved in glycotoxin detoxification and/or mediate antioxidant and anti-inflammatory responses (23, 24, 72, 90, 94).

In humans, some RCS are found to modulate transcription through the Keap1-Nrf2 pathway, which is responsible for upregulation of the transcription of target genes encoding protective enzymes against carbonyl/oxidative stress: (i) antioxidant—superoxide dismutase, catalase, glutathione-dependent peroxidase; (ii) antiglycation—glyoxalase 1; and (iii) associated with antioxidant and antiglycation—peroxiredoxin reductase, thioredoxin reductase, γ-glutamylcysteine synthase, glutathione reductase, glutathione S-transferases (83, 85, 95). Generally, a highly developed and tightly regulated antiglycoxidation system also includes such enzymes as amadoriases (deglycases), aldo- and keto-reductases, alcohol and aldehyde dehydrogenases, carbonyl reductases, cytochrome P450 family, as well as low-molecular-mass antioxidants and antiglycation agents (95–97). All these components act in concert to protect against glycotoxins in various cells and body fluids, counteracting the adverse effects of glycation products while maintaining their beneficial levels. This raises a logical question about the correlation between biological impact of glycation products and their *in vivo* concentrations.

Since dietary glycation products are a major source of the total AGE pool in the body, reducing their absorption through their degradation in the gastrointestinal tract becomes critically important for preventing potential AGE-related harmful effects.

### 1.4 Human enzymatic digestion of dietary glycotoxins

Indeed, dAGEs and their precursors are unavoidable components of our daily meals, making their complete elimination unrealistic. However, minimizing their intake and adopting healthier food preparation techniques remain effective strategies for mitigating the adverse health effects associated with AGE accumulation. It is well-established that limiting added sugars, particularly fructose, and reducing dietary glycotoxins have significant health benefits (8, 12, 98–103). For instance, diets low in added sugars and dAGEs have been shown to improve insulin sensitivity, alleviate renal dysfunction, and reduce cardiovascular risk. Numerous studies offer practical advice on lowering the intake of dietary glycotoxins and their precursors, as well as on improving cooking methods and adopting AGE-reducing eating habits (4, 12, 46, 104, 105). In addition, enhancing the digestive capacity of both human and microbial enzymes may be an effective complementary mechanism for reducing the systemic AGE burden.

Although AGE formation is a well-described process both *in vitro* and *in vivo*, the mechanisms by which the human gastrointestinal tract degrades dAGEs remain poorly investigated. It is generally recognized that AGEs are highly stable complexes and therefore tend to be accumulated in the body. The molecular mass and whether dAGEs are in a free or protein-bound form in food play a significant role in their fate in the intestine. Since a substantial portion of dAGEs in food is bound to proteins, this suggests that proteolytic digestion may play a role in their breakdown and some human digestive enzymes, such as elastase, trypsin, and chymotrypsin, are likely to contribute to the degradation of protein-bound dAGEs (105–107). On the other hand, glycation of arginine and lysine residues can block the sites of the proteases responsible for the protein digestion (44). In addition, binding of dAGEs to proteins increases their molecular mass and may therefore reduce their degradation, impairing their digestibility and absorption.

Most studies addressing this issue have been performed *in vitro*, using nonenzymatic glycation of proteins of known structure, followed by their enzymatic degradation. A commonly used experimental approach involves static and dynamic *in vitro* gastrointestinal digestion models. A wide range of food matrices has been tested in such experiments—from infant formulas, which are well-defined in their composition and rich in dAGEs, to more complex mixtures such as processed meats, biscuits, and canned foods. Despite the “simplicity” of *in vitro* models, the available studies do not yet provide a clear picture of how dAGEs can behave during digestion. Moreover, sometimes the findings are inconsistent. Some studies have reported breakdown of protein-bound dAGEs during digestion, however, most evidence suggests that a substantial fraction of these protein-bound dAGEs survive passage through the gastrointestinal tract.

Using the validated dynamic TNO gastrointestinal model, recent study has provided mechanistic insights into the digestive fate of protein-bound dAGEs conditions (105). The *in vitro* system mimics human upper gastrointestinal conditions, including dynamic pH changes, peristaltic movements, bile secretion, enzymatic activity, and passive absorption. It has been shown that hydrolysis of protein-bound dAGEs partial occurs, particularly under small-intestinal conditions. Similar findings were reported by authors, who observed that most peptide release took place in the duodenum of an *in vitro* infant gastrointestinal model (108). In addition, proteolytic susceptibility appears to vary among glycated proteins, suggesting that protein type influences digestion efficiency (109). At the same time, investigation of bioactive proteins such as lactoferrin demonstrated that mild glycation can even increase their susceptibility to proteolysis (110, 111).

Other studies investigating the *in vitro* digestion of glycated proteins demonstrated that glycation of proteins impairs their enzymatic hydrolysis, thereby reducing the subsequent absorption of peptides and amino acids (112, 113). Moreover, results from the *in vitro* simulated digestion experiments revealed that glycation decreased the content of free essential and total amino acids appear after digestion (110, 114).

Notably, several *in vitro* experiments demonstrated that additional AGEs and other glycotoxins may be formed during gastrointestinal digestion (43, 105, 110), while others do not support this (115). It is likely that the chemical conditions in the stomach, particularly the low pH, do not favor significant AGE formation at this stage of digestion. This idea has been supported by demonstration that using acidic ingredients such as lemon juice or vinegar to lower pH can help reduce formation of AGEs during cooking (12, 46, 47). Conversely, preparing food under slightly alkaline conditions enhances AGE formation.

The variety of experimental conditions, including the models used, the types of material tested, and the methods for AGE assessment largely explain different and sometimes even contradictory results. Nevertheless, *in vitro* models remain a crucial first step toward a deeper understanding of how dAGEs are digested and transformed in the human gastrointestinal tract. What is beyond doubt, is that the most ingested dAGEs survive the initial stages of digestion and absorption and therefore reach the intestine, where they interact with the gut microbiota. These microbial communities appear to represent an important link in the complex host mechanisms that prevent the detrimental effects of glycation products and may even enable the host to derive potential benefits from dietary glycotoxins.

### 1.5 Microbiome-glycotoxin interactions

The impact of glycation on microorganisms has been investigated in numerous studies, either directly or indirectly. Since the middle 1950s, early works (although without addressing the Maillard reaction) examined bacterial species such as *Bacillus subtilis, Escherichia coli*, lactic acid bacteria, and propionic acid bacteria for their growth on media, in which nitrogen and carbohydrate sources were autoclaved together (116). To this day, researchers usually grow experimental cultures on media containing both reducing carbohydrates and nitrogen sources such as amino acids, which are sterilized at high temperatures. Obviously, this sterilization process promotes glycation in the cultivation media.

A large cluster of studies demonstrates that such media affect the phenotype of microorganisms. For example, substituting glucose with fructose in an autoclaved cultivation medium influences growth, metabolic activity, and adaptation potential (117–121). One explanation for the different effects of the two monosaccharide isomers on microbial growth is their various abilities to initiate glycation both *in vitro* and *in vivo* (39, 79, 122). Incubation of fructose either with or without albumin, cell-free extracts, or cell cultures under physiological conditions leads to generation of higher concentrations of ROS, RCS, and AGEs as compared with glucose (36, 37, 39, 122). Microorganisms grown on fructose exhibit higher levels of glycoxidation products and markers of carbonyl/oxidative stress than those grown on glucose. Depending on the cultivation period on autoclaved medium, fructose supplementation led either to a more pronounced age-related decline in microbial reproductive ability and higher cell mortality (long-term model) or, conversely, to higher survival under stressful conditions (short-term model) (81, 122).

Some evidence also indicates that certain gut bacteria may themselves serve as a source of glycation products in the intestine. For instance, *Enterococcus faecalis, Escherichia coli, Pediococcus acidilactici*, and *Ruminococcus* sp. have been shown to exhibit relatively high activity in methylglyoxal formation and its excretion during bacterial growth, thereby potentially influencing the intestinal balance of glycation products in the host (32, 123–127). It should be noted that methylglyoxal is generated not only as a by-product of glycolysis but may also be formed by methylglyoxal synthase—the enzyme, which is possessed by many of gut bacteria (32, 125, 128). Although bacteria have a short lifespan and an intensive protein turnover, while the formation of glycation products is a relatively slow process, glycation products (in particular those bound to proteins) have nevertheless been detected in *Escherichia coli* cells as well as their cultivation medium (129).

Glycation agents and glycation products in growth media clearly influence the phenotype of microorganisms under laboratory conditions and their potential impact on the gut microbiome is likely to be even more complex and multifaceted. There is growing evidence that microorganisms within the colon play a key role in maintaining host health due to their essential metabolic functions, ability to modulate the immune response, and contribution to the digestion of otherwise indigestible dietary components (1, 103, 104, 130–132). In this context, the interaction between glycotoxins and gut microbiota has attracted increasing scientific interest.

Unabsorbed dietary glycotoxins can exert both beneficial and detrimental influences on the composition of the gut microbiome (44, 103). In rodent models, high-AGE diets reduced microbial diversity and the abundance of saccharolytic bacteria, while increasing potentially harmful species of *Desulfovibrio* and *Bacteroides*, and elevating toxic microbial metabolites like cresol and putrescine (133, 134). On the other hand, certain glycation products exhibit antimicrobial activity, suppressing bacteria such as *Helicobacter pylori* and *Staphylococcus aureus* (44, 135, 136). Together, these findings support suggestions that unabsorbed dietary glycotoxins can modulate the colonic microbiota with both beneficial and detrimental consequences.

Among the potential benefits of glycation products for the gut-friendly microbiome may be a stimulation of bacterial adaptive mechanisms by low doses of dietary glycotoxins. This aspect remains poorly explored; however, similar mechanisms of hormetic responses across living organisms (78, 137–139) suggest that glycotoxins exert a mild hormetic effects on the gut microbiota. Moreover, methylglyoxal, glyoxal, and other glycation agents are known to induce adaptive survival responses in microorganisms such as *E. coli, Streptococcus mutans* and *S. cerevisiae* (33, 81, 119, 140, 141). Adaptations induced by mild stress enable microorganisms to survive under severe or even lethal challenges; these adaptations are strain-specific and regulated on different levels of the genetic information flow (116, 126, 140–145). Overall, low doses of glycotoxins may induce adaptation, potentially providing benefits directly or indirectly for both the host and the gut microbiota (119, 142, 146–148).

Similar to reactive carbonyl species and other reactive glycotoxins, the influence of dAGEs on gut microbiota is suggested to depend on conditions and to vary with the ability of bacteria to metabolize these poorly degradable complexes (44, 103). The large intestine hosts a diverse community of species with various metabolic abilities that determine the fate of glycated products. Among the most studied members of the colonic microbiota are genera such as *Bacteroides, Escherichia, Bifidobacterium*, and *Lactobacillus* (4, 44, 103). The human gut microbiome has a greater diversity of degradative activities than the host, suggesting a critical role for bacterial enzymes in metabolism of glycotoxins. Unabsorbed early glycation products and high-molecular-mass dAGEs or their metabolites may serve as potential substrates for intestinal microorganisms (149–151). Bacteria possess specific enzymes capable of metabolizing glycation products, including amadoriases, glyoxalases, fructosamine kinases, and ribulosamine/erythrulosamine 3-kinases (152–154).

It has been found that colonic bacteria can degrade early glycation products. For example, *E. coli* metabolizes fructoselysine (155) and psicoselysin (156). *E. coli* also uses fructosamine kinase to convert fructosamine into fructosamine-6-phosphate, which is then transformed to glucose-6-phosphate by deglycase (157). Glucose-6-phosphate, being at the metabolic crossroads, can enter catabolic pathways to provide energy or alternatively, serve as a precursor for the synthesis of carbohydrates and amino acids. By degrading glycated amino acids, the human colonic microbiota can utilize them as sources of energy, carbon, and nitrogen (158). The distal colonic microbiota can degrade even some high-molecular-mass glycated proteins (159). Moreover, colonic bacteria are capable of metabolizing glycation products at different stages of the same pathway—from early to advanced glycation products. For instance, both fructoselysine and its derivative carboxymethyllysine can be degraded by the same human intestinal microbiota samples, although with different efficiencies: fructoselysine is degraded more readily than carboxymethyllysine (about 28-fold higher) (126, 127, 158).

Interestingly, fructoselysine can be utilized by the gut microbiota to produce butyric acid, one of the major short-chain fatty acids, that are among important groups of microbial metabolites in the human intestine. For example, an *in vitro* model demonstrated a correlation between the formation of short-chain fatty acids and fructoselysine degradation; notably, among the short-chain fatty acids measured, butyrate appeared to be the predominant product of fructoselysine breakdown (160). Among the best-studied human gut butyrate producers are *Faecalibacterium prausnitzii, Butyricicoccus pullicaecorum*, and *Subdoligranulum variabile*. These bacteria generate butyrate primarily from carbohydrates via the acetyl-CoA pathway or from amino acids via the lysine pathway, but not from fructoselysine (161). In contrast, *Bacillus subtilis* and *Escherichia coli* can degrade fructoselysine but do not produce butyrate as an end product (157).

A human gut commensal *Intestinimonas butyriciproducens* appears to be an important species capable of converting fructoselysine to butyrate through both the acetyl-CoA and lysine pathways. When growing on glucose, it utilizes the acetyl-CoA pathway, whereas during lysine fermentation it employs the lysine pathway; in both cases, fructoselysine can be converted to butyrate. In the presence of fructoselysine, *I. butyriciproducens* simultaneously uses both pathways, converting 1 mole of fructoselysine into 3 moles of butyrate (162). However, *I. butyriciproducens* is a relatively rare commensal—only about 10% of 65 fecal metagenomes from the Human Microbiome Project contained *I. butyriciproducens* AF211 genes involved in fructoselysine degradation (162, 163). This limited prevalence points to marked interindividual differences in the microbial potential for fructoselysine degradation. The authors proposed that additional, as yet unidentified gut bacteria may also contribute to this process (161).

For the host organism, these pathways are particularly important because fructoselysine—one of the most abundant Amadori products associated with metabolic and other disorders (47)—is thereby detoxified by *I. butyriciproducens*. Generaly, butyrogenic gut bacteria can utilize glycated metabolites as substrates for producing the valuable metabolites. The gut microbiome generates short-chain fatty acids, and butyrate in particular, from precursors such as lactate, succinate, certain amino acids, and carbohydrates (164–167). The generated butyrate, in turn, is a key metabolite that exerts a wide range of beneficial effects on the host, including immunoregulatory and anti-inflammatory actions, as well as anti-obesity, antidiabetic, anticancer, cardioprotective, hepatoprotective, and neuroprotective properties. In addition, butyrate contributes to intestinal health by strengthening the epithelial barrier, enhancing mucosal immunity, and reducing inflammation. It also participates in gut-brain axis signaling and displays antitumor effects by promoting apoptosis and cell cycle arrest in malignant colonocytes, while supporting proliferation in normal colonocytes (2, 4, 166–169).

The latter not only helps protect the host against harmful glycation products, contributing in particular to a healthy colon, but also it is one of the key mechanisms for maintaining a healthy gut microbiome. The overall picture of glycotoxin-microbiome interplay appears even more complex than described above when taking into account that the effects of dietary glycotoxins on the gut microbiota depend not only on the bacterial species, type of glycation products and their amounts, but also on the individual's phenotype—including age, sex, dietary habits, physical activity, and other factors (1, 68, 131, 146, 170–172).

## 2 Conclusions and perspectives

**Figure 4:**
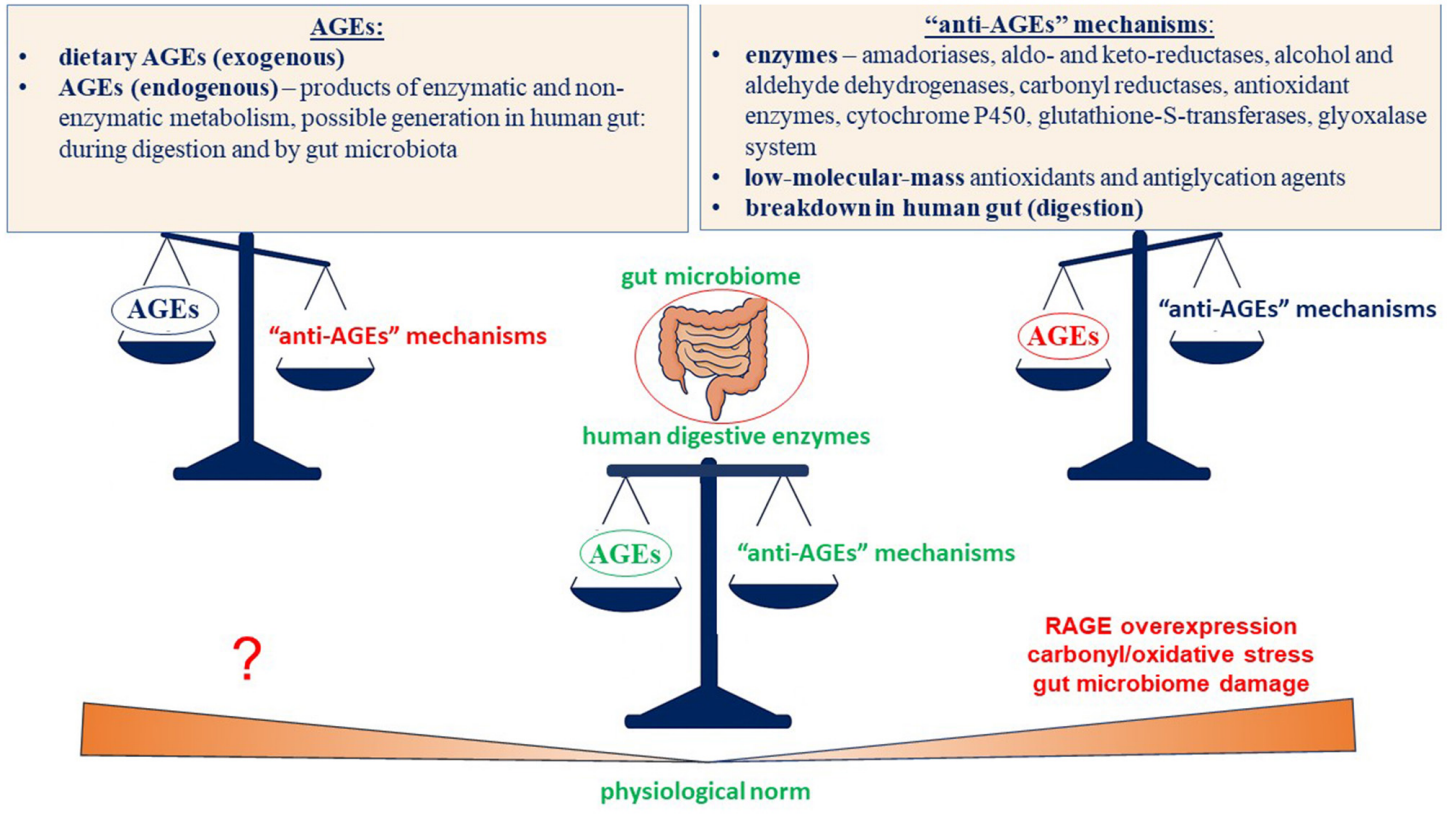
Dynamic balance between formation/intake of AGEs and their degradation: the role of human digestive enzymes and gut microbiome in maintaining physiological concentrations of glycation products. Maintaining an appropriate balance of glycotoxins depends largely on digestion in the gastrointestinal tract. Both host and microbial enzymes work in concert to degrade glycation products and maintain their concentrations within a potentially “beneficial” range. The right and left scales Illustrate imbalance scenarios: excess AGEs or insufficient degrading mechanisms (right) can shift the balance toward harmful outcomes, including RAGE overexpression, carbonyl/oxidative stress, and gut microbiome disruption, while an unexplored state of overly low AGE levels (left) raises open questions. In both cases, the complementary roles of host digestion and the gut microbiome in maintaining AGE homeostasis and preventing harmful consequences of imbalance appear to be the crucial.

Although Maillard chemistry has been extensively studied both *in vitro* and *in vivo* and numerous studies have demonstrated the detrimental effects of glycation products on human health, particularly due to: (1) RAGE overexpression, contributing to inflammation and related processes; (2) development of chronic carbonyl/oxidative stress, under which biomolecules can be irreversibly damaged; and (3) harmful impacts on the gut microbiome—many uncertainties remain (Figure 4). Most, if not all, are closely linked to following issue—what are the “normal” levels of glycotoxins in the human body? This question arises because glycation products are unavoidable—they are formed endogenously during normal metabolism and are also present in even “healthy” foods.

Over time, the human organism has adapted to glycotoxins and has even learned to use them. For example, the immune system employs reactive carbonyl metabolites as a “weapon” against pathogens. However, more remains to be clarified regarding AGEs, particularly their role in maintaining the “normal” level of RAGE expression, since this receptor fulfills important physiological functions beyond triggering harmful events. Defining the concentration ranges of glycation products that activate RAGE for its necessary functions is not simple, because the receptor is not specific to AGEs alone; it can bind multiple ligands, thereby activating a complex signaling network that remains not fully investigated. Moreover, the “negative” effects of RAGE activation cannot always be attributed solely to AGEs.

Maintaining “proper” concentrations of glycation products depends on a balance between, on the one hand, their endogenous formation and dietary intake, and on the other hand, the activity of antiglycation system and digestion of glycotoxins in the gastrointestinal tract. The functioning of the antiglycation system and its regulation are relatively well-studied, whereas the fate of glycation products in the gut remains less clear, although progress is being made. Transformation of glycation products depends greatly on the gut microbiome and its complementary functioning with host digestive enzymes. The microbiome not only degrades glycation products—influencing their absorption and entry into the bloodstream—but is itself significantly affected by glycotoxins. This raises an important issue: how are glycation products degraded within the human digestive system, and how do they influence, and, in turn, are influenced by the gut microbiome? Therefore, the question of what are “beneficial” concentrations of glycotoxins *in vivo* emerges again. It is also important because mild stress, induced by glycotoxins at low their concentrations, may actually be beneficial, stimulating bacterial defense mechanisms—a process that is critically important for strengthening and selecting essential gut-friendly bacteria. Furthermore, glycation products can serve as substrates for certain microbes, which, in turn, produce valuable metabolites for the host, such as short-chain fatty acids.

It is also worth considering the opposite scenario: what happens when the level of glycation products falls below a hypothetical physiological norm? What consequences might this have for the gastrointestinal tract, the gut microbiome, and the overall health of the host? One of the fundamental open questions is the extent to which digestive enzymes and microbial enzymes in the human body, act in a complementary manner to degrade glycation products, thereby maintaining “appropriate” concentrations in the human organism.

## References

[1] Bengmark S. Gut microbiota, immune development and function. Pharmacol Res. (2013) 69:87–113. doi: 10.1016/j.phrs.2012.09.002 22989504

[2] Bengmark S. Choose right carbohydrates and right fats (RCRF) - keys to optimal health. Hepatobiliary Surg Nutr. (2017) 6:429–33. doi: 10.21037/hbsn.2017.12.03 29312983 PMC5756773

[3] Twarda-Clapa A Olczak A Białkowska AM Koziołkiewicz M. Advanced glycation end-products (AGEs): formation, chemistry, classification, receptors, and diseases related to AGEs. Cells. (2022) 11:1312. doi: 10.3390/cells11081312 35455991 PMC9029922

[4] Kumar S Mukherjee R Gaur P Leal É Lyu X Ahmad S . Unveiling roles of beneficial gut bacteria and optimal diets for health. Front Microbiol. (2025) 16:1527755. doi: 10.3389/fmicb.2025.1527755 40041870 PMC11877911

[5] Tessier FJ. The Maillard reaction in the human body. The main discoveries and factors that affect glycation. Pathol Biol. (2010) 58:214–9. doi: 10.1016/j.patbio.2009.09.014 19896783

[6] Robert L Labat-Robert J Robert AM. The Maillard reaction. From nutritional problems to preventive medicine. Pathol Biol. (2010) 58:200–6. doi: 10.1016/j.patbio.2009.09.004 19896300

[7] Robert L Robert AM Labat-Robert J. The Maillard reaction – illicite (bio)chemistry in tissues and food. Pathol Biol. (2011) 59:321–8. doi: 10.1016/j.patbio.2011.04.007 21640521

[8] Nie C Li Y Qian H Ying H Wang L. Advanced glycation end products in food and their effects on intestinal tract. Crit Rev Food Sci Nutr. (2022) 62:3103–15. doi: 10.1080/10408398.2020.1863904 33356474

[9] Bengmark S. Advanced glycation and lipoxidation end products-amplifiers of inflammation: the role of food. J Parenter Enteral Nutr. (2007) 31:430–40. doi: 10.1177/0148607107031005430 17712153

[10] Nogueira Silva Lima MT Delayre-Orthez C Howsam M Jacolot P Niquet-Léridon C Okwieka A . Early- and life-long intake of dietary advanced glycation end-products (dAGEs) leads to transient tissue accumulation, increased gut sensitivity to inflammation, and slight changes in gut microbial diversity, without causing overt disease. Food Res Int. (2024) 195:114967. doi: 10.1016/j.foodres.2024.114967 39277266

[11] Krautwald M Münch G. Advanced glycation end products as biomarkers and gerontotoxins – a basis to explore methylglyoxal-lowering agents for Alzheimer's disease? Exp Gerontol. (2010) 45:744–51. doi: 10.1016/j.exger.2010.03.001 20211718

[12] Uribarri J del Castillo MD de la Maza MP Filip R Gugliucci A Luevano-Contreras C . Dietary advanced glycation end products and their role in health and disease. Adv Nutr. (2015) 6:461–73. doi: 10.3945/an.115.008433 26178030 PMC4496742

[13] Gu MJ Lee YR Kim D Kim Y Ha SK. Comprehensive research on the properties of advanced glycation end products in food and biological samples and their harmful role in inducing metabolic diseases. Compr Rev Food Sci Food Saf. (2024) 23:e13412. doi: 10.1111/1541-4337.13412 39137000

[14] Zinöcker MK Lindseth IA. The western diet-microbiome-host interaction and its role in metabolic disease. Nutrients. (2018) 10:365. doi: 10.3390/nu10030365 29562591 PMC5872783

[15] Petriv N Neubert L Vatashchuk M Timrott K Suo H Hochnadel I . Increase of α-dicarbonyls in liver and receptor for advanced glycation end products on immune cells are linked to nonalcoholic fatty liver disease and liver cancer. Oncoimmunology. (2021) 10:1874159. doi: 10.1080/2162402X.2021.1874159 33628620 PMC7889131

[16] Maillard LC. Action des acides amines sur les sucres: formation des melanoidines par voie Methodique. CR Acad Sci. (1912) 154:66–8. French.

[17] Hodge JE. Dehydrated foods: chemistry of browning reactions in model systems. J Agric Food Chem. (1953) 1:928–43. doi: 10.1021/jf60015a004

[18] Rahbar S. Hemoglobin H disease in two Iranian families. Clin Chim Acta. (1968) 20:381–5. doi: 10.1016/0009-8981(68)90293-3 5658939

[19] Rahbar S Blumenfeld O Ranney HM. Studies of an unusual hemoglobin in patients with diabetes mellitus. Biochem Biophys Res Commun. (1969) 36:838–43. doi: 10.1016/0006-291X(69)90685-8 5808299

[20] Yatscoff RW Tevaarwerk GJ MacDonald JC. Quantification of nonenzymically glycated albumin and total serum protein by affinity chromatography. Clin Chem. (1984) 30:446–49. doi: 10.1093/clinchem/30.3.446 6697494

[21] Monnier VM Cerami A. Nonenzymatic browning *in vivo*: possible process for aging of long-lived proteins. Science. (1981) 211:491–3. doi: 10.1126/science.6779377 6779377

[22] Cerami A. Hypothesis: glucose as amediator of aging. J Am Geriatr Soc. (1985) 33:626–34. doi: 10.1111/j.1532-5415.1985.tb06319.x 3897348

[23] Semchyshyn H. Fructose-mediated AGE-RAGE axis: approaches for mild modulation. Front Nutr. (2024) 11:1500375. doi: 10.3389/fnut.2024.1500375 39698244 PMC11652219

[24] Ott C Jacobs K Haucke E Navarrete Santos A Grune T Simm A. Role of advanced glycation end products in cellular signaling. Redox Biol. (2014) 2:411–29. doi: 10.1016/j.redox.2013.12.016 24624331 PMC3949097

[25] Nowotny K Jung T Höhn A Weber D Grune T. Advanced glycation end products and oxidative stress in type 2 diabetes mellitus. Biomolecules. (2015) 5:194–222. doi: 10.3390/biom5010194 25786107 PMC4384119

[26] Cai W Gao QD Zhu L Peppa M He C Vlassara H. Oxidative stress-inducing carbonyl compounds from common foods: novel mediators of cellular dysfunction. Mol Med. (2002) 8:337–46. doi: 10.1007/BF03402014 12393931 PMC2040002

[27] Kikuchi S Shinpo K Takeuchi M Yamagishi S Makita Z Sasaki N . Glycation-a sweet tempter for neuronal death. Brain Res Brain Res Rev. (2003) 41:306–23. doi: 10.1016/S0165-0173(02)00273-4 12663085

[28] Uribarri J Cai W Peppa M Goodman S Ferrucci L Striker G . Circulating glycotoxins and dietary advanced glycation endproducts: two links to inflammatory response, oxidative stress, and aging. J Gerontol A Biol Sci Med Sci. (2007) 62:427–33. doi: 10.1093/gerona/62.4.427 17452738 PMC2645629

[29] Peng X Ma J Chen F Wang M. Naturally occurring inhibitors against the formation of advanced glycation end-products. Food Funct. (2011) 2:289–301. doi: 10.1039/c1fo10034c 21779567

[30] Martinez Fernandez A Regazzoni L Brioschi M Gianazza E Agostoni P Aldini G . Pro-oxidant and pro-inflammatory effects of glycated albumin on cardiomyocytes. Free Radic Biol Med. (2019) 144:245–55. doi: 10.1016/j.freeradbiomed.2019.06.023 31260731

[31] Kalapos MP. Methylglyoxal and glucose metabolism: a historical perspective and future avenues for research. Drug Metabol Drug Interact. (2008) 23:69–91. doi: 10.1515/DMDI.2008.23.1-2.69 18533365

[32] Akinrimisi OI Maasen K Scheijen JLJM Nemet I Nieuwdorp M Schalkwijk CG . Does gut microbial methylglyoxal metabolism impact human physiology? Antioxidants. (2025) 14:763. doi: 10.3390/antiox14070763 40722867 PMC12291648

[33] Chakraborty S Karmakar K Chakravortty D. Cells producing their own nemesis: understanding methylglyoxal metabolism. IUBMB Life. (2014) 66:667–78. doi: 10.1002/iub.1324 25380137

[34] Martins AM Cordeiro CA Ponces Freire AM. In situ analysis of methylglyoxal metabolism in *Saccharomyces cerevisiae. FEBS Lett*. (2001) 499:41–4. doi: 10.1016/S0014-5793(01)02519-4 11418108

[35] Ramasamy R Vannucci SJ Yan SS Herold K Yan SF Schmidt AM. Advanced glycation end products and RAGE: a common thread in aging, diabetes, neurodegeneration, and inflammation. Glycobiology. (2005) 15:16R–28R. doi: 10.1093/glycob/cwi053 15764591

[36] Sakai M Oimomi M Kasuga M. Experimental studies on the role of fructose in the development of diabetic complications. Kobe J Med Sci. (2002) 48:125–36. 12594356

[37] Spasojević I Bajić A Jovanović K Spasić M Andjus P. Protective role of fructose in the metabolism of astroglial C6 cells exposed to hydrogen peroxide. Carbohydr Res. (2009) 344:1676–81. doi: 10.1016/j.carres.2009.05.023 19591975

[38] Semchyshyn H. Fructation *in vivo*: detrimental and protective effects of fructose. Biomed Res Int. (2013) 2013:343914. doi: 10.1155/2013/343914 23984346 PMC3741926

[39] Semchyshyn H Miedzobrodzki J Bayliak MM Lozinska LM Homza BV. Fructose compared with glucose is a more potent glycoxidation agent *in vitro*, but not under carbohydrate-induced stress *in vivo*: potential role of antioxidant and antiglycation enzymes. Carbohydr Res. (2014) 384:61–9. doi: 10.1016/j.carres.2013.11.015 24361593

[40] Semchyshyn H. Is part of the fructose effects on health related to increased AGE formation? In:Uribarri J, editor. Dietary AGEs and Their Role in Health and Disease. Boca Raton: CRC Press (2017). p. 103–11. doi: 10.1201/9781315120041-10

[41] Gaut JP Yeh GC Tran HD Byun J Henderson JP Richter GM . Neutrophils employ the myeloperoxidase system to generate antimicrobial brominating and chlorinating oxidants during sepsis. Proc Natl Acad Sci USA. (2001) 98:11961–6. doi: 10.1073/pnas.211190298 11593004 PMC59821

[42] Garay-Sevilla ME Rojas A Portero-Otin M Uribarri J. Dietary AGEs as exogenous boosters of inflammation. Nutrients. (2021) 13:2802. doi: 10.3390/nu13082802 34444961 PMC8401706

[43] Monteiro-Alfredo T Matafome P. Gut metabolism of sugars: formation of glycotoxins and their intestinal absorption. Diabetology. (2022) 3:596–605. doi: 10.3390/diabetology3040045

[44] Zhao D Sheng B Wu Y Li H Xu D Nian Y . Comparison of free and bound advanced glycation end products in food: a review on the possible influence on human health. J Agric Food Chem. (2019) 7:14007–18. doi: 10.1021/acs.jafc.9b05891 31789029

[45] Goldberg T Cai W Peppa M Dardaine V Baliga BS Uribarri J . Advanced glycoxidation end products in commonly consumed foods. J Am Diet Assoc. (2004) 104:1287–91. doi: 10.1016/j.jada.2004.05.214 15281050

[46] Uribarri J Woodruff S Goodman S Cai W Chen X Pyzik R . Advanced glycation end products in foods and a practical guide to their reduction in the diet. Am Diet Assoc. (2010) 110:911–16.e12. doi: 10.1016/j.jada.2010.03.018 20497781 PMC3704564

[47] Poulsen MW Hedegaard RV Andersen JM de Courten B Bügel S Nielsen J . Advanced glycation endproducts in food and their effects on health. Food Chem Toxicol. (2013) 60:10–37. doi: 10.1016/j.fct.2013.06.052 23867544

[48] Inan-Eroglu E Ayaz A Buyuktuncer Z. Formation of advanced glycation endproducts in foods during cooking process and underlying mechanisms: a comprehensive review of experimental studies. Nutr Res Rev. (2020) 33:77–89. doi: 10.1017/S0954422419000209 31699165

[49] Zhang Q Li H Zheng R Cao L Zhang S Zhang S . Comprehensive analysis of advanced glycation end-products in commonly consumed foods: presenting a database for dietary AGEs and associated exposure assessment. Food Sci Hum Well. (2024) 13:1917–28. doi: 10.26599/FSHW.2022.9250159

[50] Delgado-Andrade C Fogliano V. Dietary advanced glycosylation end-products (dAGEs) and melanoidins formed through the Maillard reaction: physiological consequences of their intake. Annu Rev Food Sci Technol. (2018) 9:271–91. doi: 10.1146/annurev-food-030117-012441 29350563

[51] Hohmann C Liehr K Henning C Fiedler R Girndt M Gebert M . Detection of free advanced glycation end products *in vivo* during hemodialysis. J Agric Food Chem. (2017) 65:930–37. doi: 10.1021/acs.jafc.6b05013 28112514

[52] Perrone A Giovino A Benny J Martinelli F. Advanced glycation end products (AGEs): biochemistry, signaling, analytical methods, and epigenetic effects. Oxid Med Cell Longev. (2020) 2020:3818196. doi: 10.1155/2020/3818196 32256950 PMC7104326

[53] Kat W Kaz W Jaramillo S Ortiz SJ Corrales Escobosa AR. What are AGEs, their chemical structure, and how can they be measured? In:Uribarri J, editor. Dietary AGEs and Their Role in Health and Disease. Boca Raton: CRC Press (2017). p. 3–17. doi: 10.1201/9781315120041-2

[54] Henle T. AGEs in foods: do they play a role in uremia? Kidney Int Suppl. (2003) S145–7. doi: 10.1046/j.1523-1755.63.s84.16.x 12694332

[55] Tuohy KM Hinton DJ Davies SJ Crabbe MJ Gibson GR Ames JM. Metabolism of Maillard reaction products by the human gut microbiota-implications for health. Mol Nutr Food Res. (2006) 50:847–57. doi: 10.1002/mnfr.200500126 16671057

[56] Portero-Otin M de la Maza MP Uribarri J. Dietary advanced glycation end products: their role in the insulin resistance of aging. Cells. (2023) 12:1684. doi: 10.3390/cells12131684 37443718 PMC10340703

[57] Huang Y Chen Z Chen B Li J Yuan X Li J . Dietary sugar consumption and health: umbrella review. BMJ. (2023) 381:e071609. doi: 10.1136/bmj-2022-071609 37019448 PMC10074550

[58] Koschinsky T He CJ Mitsuhashi T Bucala R Liu C Buenting C . Orally absorbed reactive glycation products (glycotoxins): an environmental risk factor in diabetic nephropathy. Proc Natl Acad Sci U S A. (1997) 94:6474–9. doi: 10.1073/pnas.94.12.6474 9177242 PMC21074

[59] Degen J Beyer H Heymann B Hellwig M Henle T. Dietary influence on urinary excretion of 3-deoxyglucosone and its metabolite 3-deoxyfructose. J Agric Food Chem. (2014) 62:2449–56. doi: 10.1021/jf405546q 24579887

[60] Foerster A Henle T. Glycation in food and metabolic transit of dietary AGEs (advanced glycation end-products): studies on the urinary excretion of pyrraline. Biochem Soc Trans. (2003) 31:1383–5. doi: 10.1042/bst0311383 14641068

[61] Förster A Kühne Y Henle T. Studies on absorption and elimination of dietary Maillard reaction products. Ann NY Acad Sci. (2005) 1043:474–81. doi: 10.1196/annals.1333.054 16037269

[62] Uribarri J Cai W Sandu O Peppa M Goldberg T Vlassara H. Diet-derived advanced glycation end products are major contributors to the body's AGE pool and induce inflammation in healthy subjects. Ann N Y Acad Sci. (2005) 1043:461–6. doi: 10.1196/annals.1333.052 16037267

[63] Yamagishi S Ueda S Okuda S. Food-derived advanced glycation end products (AGEs): a novel therapeutic target for various disorders. Curr Pharm Des. (2007) 13:2832–6. doi: 10.2174/138161207781757051 17897026

[64] Scheijen JLJM Hanssen NMJ van Greevenbroek MM Van der Kallen CJ Feskens EJM Stehouwer CDA . Dietary intake of advanced glycation endproducts is associated with higher levels of advanced glycation end products in plasma and urine: the CODAM study. Clin Nutr. (2018) 37:919–25. doi: 10.1016/j.clnu.2017.03.019 29381139

[65] Krajcovicová-Kudlácková M Sebeková K Schinzel R Klvanová J. Advanced glycation end products and nutrition. Physiol Res. (2002) 51:313–6. doi: 10.33549/physiolres.930200 12234125

[66] Šebekova K Brouder Šebekova K. Dietary AGEs may have different effects in people with vegetarian versus omnivorous eating patterns. In:Uribarri J, editor. Dietary AGEs and Their Role in Health and Disease. Boca Raton: CRC Press (2017). p. 225–37. doi: 10.1201/9781315120041-21

[67] Pérez-Burillo S Pastoriza S Rufian-Henares JÁ. Effects of dietary AGEs in the gut microbiota composition. In:Uribarri J, editor. Dietary AGEs and Their Role in Health and Disease. Boca Raton: CRC Press (2017). p. 239–45. doi: 10.1201/9781315120041-22

[68] Chen Y Guo TL. Dietary advanced glycation end-products elicit toxicological effects by disrupting gut microbiome and immune homeostasis. J Immunotoxicol. (2021) 18:93–104. doi: 10.1080/1547691X.2021.1959677 34436982 PMC9885815

[69] Dong H Zhang Y Huang Y Deng H. Pathophysiology of RAGE in inflammatory diseases. Front Immunol. (2022) 13:931473. doi: 10.3389/fimmu.2022.931473 35967420 PMC9373849

[70] Schmidt AM Yan SD Yan SF Stern DM. The multiligand receptor RAGE as a progression factor amplifying immune and inflammatory responses. J Clin Invest. (2001) 108:949–55. doi: 10.1172/JCI14002 11581294 PMC200958

[71] Daffu G del Pozo CH O'Shea KM Ananthakrishnan R Ramasamy R Schmid AM. Radical roles for RAGE in the pathogenesis of oxidative stress in cardiovascular diseases and beyond. Int J Mol Sci. (2013) 14:19891–910. doi: 10.3390/ijms141019891 24084731 PMC3821592

[72] Ramasamy R Yan SF Schmidt AM. Receptor for AGE (RAGE): signaling mechanisms in the pathogenesis of diabetes and its complications. Ann N Y Acad Sci. (2011) 1243:88–102. doi: 10.1111/j.1749-6632.2011.06320.x 22211895 PMC4501013

[73] Gasparotto J Somensi N Girardi CS Bittencourt RR de Oliveira LM Hoefel LP . Is it all the RAGE? Defining the role of the receptor for advanced glycation end products in Parkinson's disease. J Neurochem. (2024) 168:1608–24. doi: 10.1111/jnc.15890 37381043

[74] Sorci G Riuzzi F Giambanco I Donato R. RAGE in tissue homeostasis, repair and regeneration. Biochim Biophys Acta. (2013) 1833:101–9. doi: 10.1016/j.bbamcr.2012.10.021 23103427

[75] Nitti M Marengo B Furfaro AL Pronzato MA Marinari UM Domenicotti C . Hormesis and oxidative distress: pathophysiology of reactive oxygen species and the open question of antioxidant modulation and supplementation. Antioxidants. (2022) 11:1613. doi: 10.3390/antiox11081613 36009331 PMC9405171

[76] Kim D Choi K-N Park J-I Kim E-H Majid A Bae O-N. Role of advanced glycation end products and mitohormesis in cancer development and progression. Antioxidants. (2025) 14:1165. doi: 10.3390/antiox14101165 41154474 PMC12561643

[77] Nokin MJ Durieux F Bellier J Peulen O Uchida K Spiegel DA . Hormetic potential of methylglyoxal, a side-product of glycolysis, in switching tumours from growth to death. Sci Rep. (2017) 7:11722. doi: 10.1038/s41598-017-12119-7 28916747 PMC5600983

[78] Mattson MP. Hormesis defined. Ageing Res Rev. (2008) 7:1–7. doi: 10.1016/j.arr.2007.08.007 18162444 PMC2248601

[79] Valishkevych BV Vasylkovska R Lozinska LM Semchyshyn HM. Fructose-induced carbonyl/oxidative stress in *S. cerevisiae*: involvement of TOR. Biochem Res Int. (2016) 2016:8917270. doi: 10.1155/2016/8917270 27019749 PMC4785243

[80] Semchyshyn HM. Hormetic concentrations of hydrogen peroxide but not ethanol induce cross-adaptation to different stresses in budding yeast. Int J Microbiol. (2014) 2014:485792. doi: 10.1155/2014/485792 24669223 PMC3942194

[81] Semchyshyn H Lozinska L. Fructose protects baker's yeast against peroxide stress: potential role of catalase and superoxide dismutase. FEMS Yeast Res. (2012) 12:761–73. doi: 10.1111/j.1567-1364.2012.00826.x 22741594

[82] Mavric E Wittmann S Barth G Henle T. Identification and quantification of methylglyoxal as the dominant antibacterial constituent of Manuka (*Leptospermum scoparium*) honeys from New Zealand. Mol Nutr Food Res. (2008) 2:483–89. doi: 10.1002/mnfr.200700282 18210383

[83] Xue M Rabbani N Momiji H Imbasi P Anwar MM Kitteringham N . Transcriptional control of glyoxalase 1 by Nrf2 provides a stress-responsive defence against dicarbonyl glycation. Biochem J. (2012) 443:213–22. doi: 10.1042/BJ20111648 22188542

[84] Semchyshyn H. Reactive carbonyl species *in vivo*: generation and dual biological effects. Sci World J. (2014) 2014:417842. doi: 10.1155/2014/417842 24634611 PMC3918703

[85] Jung KA Choi BH Nam CW Song M Kim ST Lee JY . Identification of aldo-keto reductases as NRF2-target marker genes in human cells. Toxicol Lett. (2013) 218:39–49. doi: 10.1016/j.toxlet.2012.12.026 23305850

[86] Forman HJ. Reactive oxygen species and α,β-unsaturated aldehydes as second messengers in signal transduction. Ann N Y Acad Sci. (2010) 1203:35–44. doi: 10.1111/j.1749-6632.2010.05551.x 20716281 PMC4226340

[87] Chegão A Guarda M Alexandre BM Shvachiy L Temido-Ferreira M Marques-Morgado I . Glycation modulates glutamatergic signaling and exacerbates Parkinson's disease-like phenotypes. NPJ Parkinsons Dis. (2022) 8:51. doi: 10.1038/s41531-022-00314-x 35468899 PMC9038780

[88] Khan MI Ashfaq F Alsayegh AA Hamouda A Khatoon F Altamimi TN . Advanced glycation end product signaling and metabolic complications: dietary approach. World J Diabetes. (2023) 14:995–1012. doi: 10.4239/wjd.v14.i7.995 37547584 PMC10401445

[89] Rabbani N Xue M Thornalley PJ. Dicarbonyls and glyoxalase in disease mechanisms and clinical therapeutics. Glycoconj J. (2016) 33:513–25. doi: 10.1007/s10719-016-9705-z 27406712 PMC4975768

[90] Semchyshyn H. Is carbonyl/AGE/RAGE stress a hallmark of the brain aging? Pflugers Arch. (2021) 473:723–34. doi: 10.1007/s00424-021-02529-y 33742308

[91] Pamplona R. Membrane phospholipids, lipoxidative damage and molecular integrity: a causal role in aging and longevity. Biochim Biophys Acta. (2008) 1777:1249–62. doi: 10.1016/j.bbabio.2008.07.003 18721793

[92] Jové M Pradas I Dominguez-Gonzalez M Ferrer I Pamplona R. Lipids and lipoxidation in human brain aging. Mitochondrial ATP-synthase as a key lipoxidation target. Redox Biol. (2019) 23:101082. doi: 10.1016/j.redox.2018.101082 30635167 PMC6859548

[93] Rabbani N Thornalley PJ. Dicarbonyls (glyoxal, methylglyoxal, and 3-deoxyglucosone). In:Niwa T, editor. Uremic Toxins. Hoboken, NJ: John Wiley & Sons, Inc. (2012). p. 177–92. doi: 10.1002/9781118424032.ch12

[94] Prasad K Mishra M. AGE-RAGE stress, stressors, and antistressors in health and disease. Int J Angiol. (2018) 27:1–12. doi: 10.1055/s-0037-1613678 29483760 PMC5825221

[95] Garaschuk O Semchyshyn HM Lushchak VI. Healthy brain aging: interplay between reactive species, inflammation and energy supply. Ageing Res Rev. (2018) 43:26–45. doi: 10.1016/j.arr.2018.02.003 29452266

[96] Picklo MJ Olson SJ Markesbery WR Montine TJ. Expression and activities of aldo-keto oxidoreductases in Alzheimer disease. J Neuropathol Exp Neurol. (2001) 60:686–95. doi: 10.1093/jnen/60.7.686 11444797

[97] Xue M Rabbani N Thornalley PJ. Glyoxalase in ageing. Semin Cell Dev Biol. (2011) 22:293–301. doi: 10.1016/j.semcdb.2011.02.013 21320620

[98] Uribarri J Peppa M Cai W Goldberg T Lu M He C . Restriction of dietary glycotoxins reduces excessive advanced glycation end products in renal failure patients. J Am Soc Nephrol. (2003) 14:728–31. doi: 10.1097/01.asn.0000051593.41395.b9 12595509

[99] de Courten B de Courten MP Soldatos G Dougherty SL Straznicky N Schlaich M . Diet low in advanced glycation end products increases insulin sensitivity in healthy overweight individuals: a double-blind, randomized, crossover trial. Am J Clin Nutr. (2016) 103:1426–33. doi: 10.3945/ajcn.115.125427 27030534

[100] Schwarz JM Noworolski SM Erkin-Cakmak A Korn NJ Wen MJ Tai VW . Effects of dietary fructose restriction on liver fat, *de novo* lipogenesis, and insulin kinetics in children with obesity. Gastroenterology. (2017) 153:743–52. doi: 10.1053/j.gastro.2017.05.043 28579536 PMC5813289

[101] Matafome P. Another player in the field: involvement of glycotoxins and glycosative stress in insulin secretion and resistance. Diabetology. (2020) 1:24–36. doi: 10.3390/diabetology1010004

[102] Baye E Kiriakova V Uribarri J Moran LJ de Courten B. Consumption of diets with low advanced glycation end products improves cardiometabolic parameters: meta-analysis of randomised controlled trials. Sci Rep. (2017) 7:2266. doi: 10.1038/s41598-017-02268-0 28536448 PMC5442099

[103] Snelson M Coughlan MT. Dietary advanced glycation end products: digestion, metabolism and modulation of gut microbial ecology. Nutrients. (2019) 11:215. doi: 10.3390/nu11020215 30678161 PMC6413015

[104] Bengmark S. Dietary intake of AGEs and ALEs and inflammation: nutritional aspects. In:Uribarri J, editor. Dietary AGEs and Their Role in Health and Disease. Boca Raton: CRC Press (2017). p. 309–27.

[105] van der Lugt T Venema K van Leeuwen S Vrolijk MF Opperhuizen A Bast A. Gastrointestinal digestion of dietary advanced glycation end products using an *in vitro* model of the gastrointestinal tract (TIM-1). Food Funct. (2020) 11:6297–307. doi: 10.1039/d0fo00450b 32602872

[106] Xu D Li L Zhang X Yao H Yang M Gai Z . Degradation of peptide-bound Maillard reaction products in gastrointestinal digests of glyoxal-glycated casein by human colonic microbiota. J Agric Food Chem. (2019) 67:12094–104. doi: 10.1021/acs.jafc.9b03520 31566978

[107] Zhao D Li L Le TT Larsen LB Su G Liang Y . Digestibility of glyoxal-glycated beta-casein and beta-lactoglobulin and distribution of peptide-bound advanced glycation end products in gastrointestinal digests. J Agric Food Chem. (2017) 65:5778–88. doi: 10.1021/acs.jafc.7b01951 28653535

[108] Joubran Y Moscovici A Portmann R Lesmes U. Implications of the Maillard reaction on bovine alpha-lactalbumin and its proteolysis during *in vitro* infant digestion. Food Funct. (2017) 8:2295–308. doi: 10.1039/c7fo00588a 28589996

[109] Corzo-Martinez M Avila M Moreno FJ Requena T Villamiel M. Effect of milk protein glycation and gastrointestinal digestion on the growth of bifidobacteria and lactic acid bacteria. Int J Food Microbiol. (2012) 153:420–7. doi: 10.1016/j.ijfoodmicro.2011.12.006 22225833

[110] Martinez-Saez N Fernandez-Gomez B Cai W Uribarri J Del Castillo MD. *In vitro* formation of Maillard reaction products during simulated digestion of meal-resembling systems. Food Res Int. (2019) 118:72–80. doi: 10.1016/j.foodres.2017.09.056 30898355

[111] Moscovici AM Joubran Y Briard-Bion V Mackie A Dupont D Lesmes U. The impact of the Maillard reaction on the *in vitro* proteolytic breakdown of bovine lactoferrin in adults and infants. Food Funct. (2014) 5:1898–908. doi: 10.1039/c4fo00248b 24947428

[112] Pinto MS Léonil J Henry G Cauty C Carvalho AÔF Bouhallab S. Heating and glycation of β-lactoglobulin and β-casein: aggregation and *in vitro* digestion. Food Res Int. (2014) 55:70–6. doi: 10.1039/d1fo02619d

[113] Yang M Liu J Yang X Li S Li C Liu B . Effect of glycation degree on the *in vitro* simulated gastrointestinal digestion: a promising formulation for egg white gel with controlled digestibility. Food Chem. (2021) 349:129096. doi: 10.1016/j.foodchem.2021.129096 33561796

[114] Yang Q Wang Y Yang M Liu X Lyu S Liu B . Effect of glycation degree on the structure and digestion properties of ovalbumin: a study of amino acids and peptides release after *in vitro* gastrointestinal simulated digestion. Food Chem. (2022) 373(PtB):131331. doi: 10.1016/j.foodchem.2021.131331 34731794

[115] Yuan X Feng S Li J Guo R Nie C Zhai R . Generation of advanced glycation end products from glycated protein or fructose/glyoxal-protein adducts under *in vitro* simulated gastrointestinal digestion. Food Chem. (2025) 463:141175. doi: 10.1016/j.foodchem.2024.141175 39278073

[116] Helou C Marier D Jacolot P Abdennebi-Najar L Niquet-Léridon C Tessier FJ . Microorganisms and Maillard reaction products: a review of the literature and recent findings. Amino Acids. (2014) 46:267–77. doi: 10.1007/s00726-013-1496-y 23588491

[117] Vasylkovska R Petriv N Semchyshyn H. Carbon sources for yeast growth as a precondition of hydrogen peroxide induced hormetic phenotype. Int J Microbiol. (2015) 2015:697813. doi: 10.1155/2015/697813 26843865 PMC4710903

[118] Semchyshyn H Valishkevych BV. Hormetic effect of H_2_O_2_ in *Saccharomyces cerevisiae*: involvement of TOR and glutathione reductase. Dose Response. (2016) 14:1559325816636130. doi: 10.1177/1559325816636130 27099601 PMC4822199

[119] Semchyshyn H. Reactive carbonyls induce TOR- and carbohydrate-dependent hormetic response in yeast. Sci World J. (2020) 2020:4275194. doi: 10.1155/2020/4275194 32231465 PMC7091552

[120] Garnett JP Braun D McCarthy AJ Farrant MR Baker EH Lindsay JA . Fructose transport-deficient *Staphylococcus aureus* reveals important role of epithelial glucose transporters in limiting sugar-driven bacterial growth in airway surface liquid. Cell Mol Life Sci. (2014) 71:4665–73. doi: 10.1007/s00018-014-1635-y 24810961 PMC4232747

[121] Zhang X Monnoye M Mariadassou M Beguet-Crespel F Lapaque N Heberden C . Glucose but not fructose alters the intestinal paracellular permeability in association with gut inflammation and dysbiosis in mice. Front Immunol. (2021) 12:742584. doi: 10.3389/fimmu.2021.742584 35024040 PMC8744209

[122] Semchyshyn H Lozinska LM Miedzobrodzki J Lushchak VI. Fructose and glucose differentially affect aging and carbonyl/oxidative stress parameters in *Saccharomyces cerevisiae* cells. Carbohydr Res. (2011) 346:933–8. doi: 10.1016/j.carres.2011.03.005 21459368

[123] Baskaran S Rajan DP Balasubramanian KA. Formation of methylglyoxal by bacteria isolated from human faeces. J Med Microbiol. (1989) 28:211–5. doi: 10.1099/00222615-28-3-211 2926792

[124] Srebreva LN Stoynev GA Ivanov IG. Evidence for excretion of glycation agents from *E. Coli* cells during growth. Biotechnol Biotechnol Equip. (2009) 23:1068–71. doi: 10.1080/13102818.2009.10817614

[125] Tirelli E Pucci M Squillario M Bignotti G Messali S Zini S . Effects of methylglyoxal on intestine and microbiome composition in aged mice. Food Chem Toxicol. (2025) 197:115276. doi: 10.1016/j.fct.2025.115276 39863075

[126] van Dongen KCW Ioannou A Wesseling S Beekmann K Belzer C. Differences in gut microbial fructoselysine degradation activity between breast-fed and formula-fed infants. FEMS Microbiol Ecol. (2022) 99:fiac145. doi: 10.1093/femsec/fiac145 36442156 PMC9749803

[127] van Dongen KCW Belzer C Bakker W Rietjens IMCM Beekmann K. Inter- and intraindividual differences in the capacity of the human intestinal microbiome in fecal slurries to metabolize fructoselysine and carboxymethyllysine. J Agric Food Chem. (2022) 70:11759–68. doi: 10.1021/acs.jafc.2c05756 36069406 PMC9501902

[128] Zhao C Dong H Zhang Y Li Y. Discovery of potential genes contributing to the biosynthesis of short-chain fatty acids and lactate in gut microbiota from systematic investigation in *E. coli*. NPJ Biofilms Microbiomes. (2019) 5:19. doi: 10.1038/s41522-019-0092-7 31312512 PMC6626047

[129] Mironova R Niwa T Hayashi H Dimitrova R Ivanov I. Evidence for non-enzymatic glycosylation in *Escherichia coli*. Mol Microbiol. (2001) 39:1061–68. doi: 10.1046/j.1365-2958.2001.02304.x 11251824

[130] Vaiserman A Koliada A Lushchak O. Developmental programming of aging trajectory. Ageing Res Rev. (2018) 47:105–22. doi: 10.1016/j.arr.2018.07.007 30059788

[131] Dmytriv TR Storey KB Lushchak VI. Intestinal barrier permeability: the influence of gut microbiota, nutrition, and exercise. Front Physiol. (2024) 15:1380713. doi: 10.3389/fphys.2024.1380713 39040079 PMC11260943

[132] van der Lugt T Opperhuizen A Bast A Vrolijk MF. Dietary advanced glycation endproducts and the gastrointestinal tract. Nutrients. (2020) 12:2814. doi: 10.3390/nu12092814 32937858 PMC7551018

[133] Qu W Yuan X Zhao J Zhang Y Hu J Wang J . Dietary advanced glycation end products modify gut microbial composition and partially increase colon permeability in rats. Mol Nutr Food Res. (2017) 61:1700118. doi: 10.1002/mnfr.201700118 28621836

[134] Qu W Nie C Zhao J Ou X Zhang Y Yang S . Microbiome-metabolomics analysis of the impacts of long-term dietary advanced-glycation-end-product consumption on C57BL/6 mouse fecal microbiota and metabolites. J Agric Food Chem. (2018) 66:8864–75. doi: 10.1021/acs.jafc.8b01466 30037223

[135] Trang VT Son VH Thanh LX Sarter S Shimamura T Uked H . Functional properties of Maillard reaction products in food: antimicrobial activity of aminoreductone against pathogenic bacteria. Food Sci Technol Res. (2013) 19:833–41. doi: 10.3136/fstr.19.833

[136] Hiramoto S Itoh K Shizuuchi S Kawachi Y Morishita Y Nagase Y . Melanoidin, a food proteinderived advanced Maillard reaction product, suppresses *Helicobacter pylori in vitro* and *in vivo*. Helicobacter. (2004) 9:429–35. doi: 10.1111/j.1083-4389.2004.00263.x 15361082

[137] Calabrese EJ Baldwin LA. Defining hormesis. Hum Exp Toxicol. (2002) 21:91–7. doi: 10.1191/0960327102ht217oa 12102503

[138] Lushchak VI. Dissection of the hormetic curve: analysis of components and mechanisms. Dose Response. (2014) 12:466–79. doi: 10.2203/dose-response.13-051.Lushchak 25249836 PMC4146335

[139] Calabrese EJ Nascarella M Pressman P Hayes AW Dhawan G Kapoor R . Hormesis determines lifespan. Ageing Res Rev. (2024) 94:102181. doi: 10.1016/j.arr.2023.102181 38182079

[140] Lee C Park C. Bacterial responses to glyoxal and methylglyoxal: reactive electrophilic species. Int J Mol Sci. (2017) 18:169. doi: 10.3390/ijms18010169 28106725 PMC5297802

[141] Walker AR Pham DN Noeparvar P Peterson AM Lipp MK Lemos JA . Fructose activates a stress response shared by methylglyoxal and hydrogen peroxide in *Streptococcus mutans*. mBio. (2025) 16:e0048525. doi: 10.1128/mbio.00485-25 40243330 PMC12077213

[142] Semchyshyn H Lushchak V Storey K. Possible reasons for difference in sensitivity to oxygen of two *Escherichia coli* strains. Biochemistry. (2005) 70:424–31. doi: 10.1007/s10541-005-0132-1 15892608

[143] Bayliak M Semchyshyn H Lushchak V. Effect of hydrogen peroxide on antioxidant enzyme activities in *Saccharomyces cerevisiae* is strain-specific. Biochemistry. (2006) 71:1013–20. doi: 10.1134/S0006297906090100 17009956

[144] Bayliak M Semchyshyn H Lushchak V. Possible accumulation of non-active molecules of catalase and superoxide dismutase in *S. cerevisiae* cells under hydrogen peroxide induced stress. Cent Eur J Biol. (2007) 2:326–36. doi: 10.2478/s11535-007-0021-2

[145] Semchyshyn H. Hydrogen peroxide-induced response in *E. coli and S cerevisiae*: different stages of the flow of the genetic information. Open Life Sci. (2009) 4:142–53. doi: 10.2478/s11535-009-0005-5

[146] Helou C Denis S Spatz M Marier D Rame V Alric M . Insights into bread melanoidins: fate in the upper digestive tract and impact on the gut microbiota using *in vitro* systems. Food Funct. (2015) 6:3737–45. doi: 10.1039/C5FO00836K 26364594

[147] Zemva J Fink CA Fleming TH Schmidt L Loft A Herzig S . Hormesis enables cells to handle accumulating toxic metabolites during increased energy flux. Redox Biol. (2017) 13:674–86. doi: 10.1016/j.redox.2017.08.007 28826004 PMC5565788

[148] Zemva J Pfaff D Groener JB Fleming T Herzig S Teleman A . Effects of the reactive metabolite methylglyoxal on cellular signalling, insulin action and metabolism - what we know in mammals and what we can learn from yeast. Exp Clin Endocrinol Diabetes. (2019) 127:203–14. doi: 10.1055/s-0043-122382 29421830

[149] Shi A Ji X Li W Dong L Wu Y Zhang Y . The interaction between human microbes and advanced glycation end products: the role of *Klebsiella* X15 on advanced glycation end products' degradation. Nutrients. (2024) 16:754. doi: 10.3390/nu16050754 38474882 PMC10933965

[150] Hellwig M Auerbach C Müller N Samuel P Kammann S Beer F . Metabolization of the advanced glycation end product N-ε-carboxymethyllysine (CML) by different probiotic *E. coli* strains. J Agric Food Chem. (2019) 67:1963–72. doi: 10.1021/acs.jafc.8b06748 30701968

[151] Bui TPN Troise AD Fogliano V de Vos WM. Anaerobic degradation of *N*-ε-carboxymethyllysine, a major glycation end-product, by human intestinal bacteria. J Agric Food Chem. (2019) 67:6594–602. doi: 10.1021/acs.jafc.9b02208 31091091 PMC6566499

[152] Gemayel R Fortpied J Rzem R Vertommen D Veiga-da-Cunha M Van Schaftingen E. Many fructosamine 3-kinase homologues in bacteria are ribulosamine/erythrulosamine 3-kinases potentially involved in protein deglycation. FEBS J. (2007) 274:4360–74. doi: 10.1111/j.1742-4658.2007.05948.x 17681011

[153] Suttisansanee U Honek JF. Bacterial glyoxalase enzymes. Semin Cell Dev Biol. (2011) 22:285–92. doi: 10.1016/j.semcdb.2011.02.004 21310258

[154] Van Schaftingen E Collard F Wiame E Veiga-da-Cunha M. Enzymatic repair of Amadori products. Amino Acids. (2012) 42:1143–50. doi: 10.1007/s00726-010-0780-3 20967558

[155] Wiame E Delpierre G Collard F Van Schaftingen E. Identification of a pathway for the utilization of the Amadori product fructoselysine in *Escherichia coli*. J Biol Chem. (2002) 277:42523–9. doi: 10.1074/jbc.M200863200 12147680

[156] Wiame E Van Schaftingen E. Fructoselysine 3-epimerase, an enzyme involved in the metabolism of the unusual Amadori compound psicoselysine in *Escherichia coli*. Biochem J. (2004) 378:1047–52. doi: 10.1042/bj20031527 14641112 PMC1224009

[157] Deppe VM Bongaerts J O'Connell T Maurer KH Meinhardt F. Enzymatic deglycation of Amadori products in bacteria: mechanisms, occurrence and physiological functions. Appl Microbiol Biotechnol. (2011) 90:399–406. doi: 10.1007/s00253-010-3083-4 21347729

[158] Hellwig M Bunzel D Huch M Franz CM Kulling SE Henle T. Stability of individual Maillard reaction products in the presence of the human colonic microbiota. J Agric Food Chem. (2015) 63:6723–30. doi: 10.1021/acs.jafc.5b01391 26186075

[159] Smith EA Macfarlane GT. Dissimilatory amino acid metabolism in human colonic bacteria. Anaerobe. (1997) 3:327–37. doi: 10.1006/anae.1997.0121 16887608

[160] van Dongen KCW van der Zande M Bruyneel B Vervoort JJM Rietjens IMCM Belzer C . An *in vitro* model for microbial fructoselysine degradation shows substantial interindividual differences in metabolic capacities of human fecal slurries. Toxicol In Vitro. (2021) 72:105078. doi: 10.1016/j.tiv.2021.105078 33429044

[161] Bui TP Ritari J Boeren S de Waard P Plugge CM de Vos WM. Production of butyrate from lysine and the Amadori product fructoselysine by a human gut commensal. Nat Commun. (2015) 6:10062. doi: 10.1038/ncomms10062 26620920 PMC4697335

[162] Rampanelli E Romp N Troise AD Ananthasabesan J Wu H Gül IS . Gut bacterium intestinimonas butyriciproducens improves host metabolic health: evidence from cohort and animal intervention studies. Microbiome. (2025) 13:15. doi: 10.1186/s40168-024-02002-9 39833973 PMC11744835

[163] Huttenhower C Gevers D Knight R Abubucker S Badger JH Chinwalla AT . Structure, function and diversity of the healthy human microbiome. Nature. (2012) 486:207–14. doi: 10.1038/nature11234 22699609 PMC3564958

[164] Barcenilla A Pryde SE Martin JC Duncan SH Stewart CS Henderson C . Phylogenetic relationships of butyrate-producing bacteria from the human gut. Appl Environ Microbiol. (2000) 66:1654–61. doi: 10.1128/AEM.66.4.1654-1661.2000 10742256 PMC92037

[165] Belenguer A Duncan SH Calder AG Holtrop G Louis P Lobley GE . Two routes of metabolic cross-feeding between bifidobacterium adolescentis and butyrate-producing anaerobes from the human gut. Appl Environ Microbiol. (2006) 72:3593–9. doi: 10.1128/AEM.72.5.3593-3599.2006 16672507 PMC1472403

[166] Louis P Flint HJ. Formation of propionate and butyrate by the human colonic microbiota. Environ Microbiol. (2017) 19:29–41. doi: 10.1111/1462-2920.13589 27928878

[167] Portincasa P Bonfrate L Vacca M De Angelis M Farella I Lanza E . Gut microbiota and short chain fatty acids: implications in glucose homeostasis. Int J Mol Sci. (2022) 23:1105. doi: 10.3390/ijms23031105 35163038 PMC8835596

[168] Xiong RG Zhou DD Wu SX Huang SY Saimaiti A Yang Z . Health benefits and side effects of short-chain fatty acids. Foods. (2022) 11:2863. doi: 10.3390/foods11182863 36140990 PMC9498509

[169] Singh V Lee G Son H Koh H Kim ES Unno T . Butyrate producers, “the sentinel of gut”: their intestinal significance with and beyond butyrate, and prospective use as microbial therapeutics. Front Microbiol. (2023) 13:1103836. doi: 10.3389/fmicb.2022.1103836 36713166 PMC9877435

[170] Koliada A Moseiko V Romanenko M Lushchak O Kryzhanovska N Guryanov V . Sex differences in the phylum-level human gut microbiota composition. BMC Microbiol. (2021) 21:131. doi: 10.1186/s12866-021-02198-y 33931023 PMC8088078

[171] Torquati L Gajanand T Cox ER Willis CRG Zaugg J Keating SE . Effects of exercise intensity on gut microbiome composition and function in people with type 2 diabetes. Eur J Sport Sci. (2023) 23:530–41. doi: 10.1080/17461391.2022.2035436 35107058

[172] Mastrocola R Collotta D Gaudioso G Le Berre M Cento AS Ferreira Alves G . Effects of exogenous dietary advanced glycation end products on the cross-talk mechanisms linking microbiota to metabolic inflammation. Nutrients. (2020) 12:2497. doi: 10.3390/nu12092497 32824970 PMC7551182

